# Intensive care unit-acquired hyponatremia in critically ill medical patients

**DOI:** 10.1186/s12967-020-02443-4

**Published:** 2020-07-02

**Authors:** Jae Kyeom Sim, Ryoung-Eun Ko, Soo Jin Na, Gee Young Suh, Kyeongman Jeon

**Affiliations:** 1 Department of Critical Care Medicine, Samsung Medical Center, Sungkyunkwan University School of Medicine, 81 Irwon-ro, Gangnam-gu, Seoul, 06351 Republic of Korea; 2 Division of Pulmonary, Allergy and Critical Care Medicine, Department of Internal Medicine, Korea University Guro Hospital, Korea University College of Medicine, Seoul, Republic of Korea; 3 Division of Pulmonary and Critical Care Medicine, Department of Medicine, Samsung Medical Center, Sungkyunkwan University School of Medicine, 81 Irwon-ro, Gangnam-gu, Seoul, 06351 Republic of Korea

**Keywords:** Acute kidney injury, Critical care, Diseases category, Hyponatremia, Intensive care unit

## Abstract

**Background:**

Previous research has focused on intensive care unit (ICU)-acquired hypernatremia; however, ICU-acquired hyponatremia has frequently been overlooked and has rarely been studied in surgical or mixed ICUs. The aim of this study is to investigate the incidence of ICU-acquired hyponatremia, the risk factors associated with its development, and its impact on outcomes in critically ill medical patients.

**Methods:**

We conducted a retrospective cohort study based on the prospective registry of all critically ill patients admitted to the medical ICU from January 2015 to December 2018. Baseline characteristics and management variables were compared between ICU-acquired hyponatremia and normonatremia patients.

**Results:**

Of 1342 patients with initial normonatremia, ICU-acquired hyponatremia developed in 217 (16.2%) patients and ICU-acquired hypernatremia developed in 117 (8.7%) patients. The Sequential Organ Failure Assessment (8.0 vs 7.0, *P* = 0.009) and Simplified Acute Physiology Score 3 scores (55.0 vs 51.0, *P* = 0.005) were higher in ICU-acquired hyponatremia patients compared with normonatremia patients. Baseline sodium (137.0 mmol/L vs 139.0 mmol/L, P < 0.001), potassium (4.2 mmol/L vs 4.0 mmol/L, *P* = 0.001), and creatinine (0.98 mg/dL vs 0.88 mg/dL, *P* = 0.034) levels were different between the two groups. Net volume balance over first 3 days was higher in ICU-acquired hyponatremia patients (19.4 mL/kg vs 11.5 mL/kg, *P* = 0.004) and was associated with the development of ICU-acquired hyponatremia (adjusted odds ratio, 1.004; 95% confidence interval, 1.002–1.007; *P* = 0.001). ICU mortality was similar in both groups (15.2% vs. 14.4%, *P* = 0.751), but renal replacement therapy was more commonly required in ICU-acquired hyponatremia patients (13.4% vs 7.4%, *P* = 0.007).

**Conclusions:**

ICU-acquired hyponatremia is not uncommon in critically ill medical patients. Increased volume balance is associated with its development. ICU-acquired hyponatremia is related to increased use of renal replacement therapy but not to mortality.

## Background

Hyponatremia, defined as a serum sodium concentration less than 135 mmol/L, is a common electrolyte abnormality found in critically ill patients [1]. More than one-sixth to one-fourth of patients have hyponatremia on intensive care unit (ICU) admission, and these patients have increased risk of mortality and prolonged ICU length of stay (LOS) [2–4]. Indeed, sodium level is a significant prognostic factor and is included in ICU scoring systems [5, 6]. These previous studies evaluated hyponatremia at the time of ICU admission, although new-onset hyponatremia is easily detected in clinical practice.

ICU-acquired dysnatremia is the disturbance of sodium level in patients whose sodium concentration was initially normal on ICU admission. Traditionally, research on ICU-acquired dysnatremia have focused on hypernatremia because hypernatremia in critically ill patients is usually developed after admission rather than being present at the time of admission [7, 8]. In contrast, ICU-acquired hyponatremia has frequently been overlooked. A few studies have revealed that ICU-acquired hyponatremia is not uncommon and has been observed to affect critically ill patients at a rate of 1 in 9 or higher. It is also associated with increased hospital mortality [4, 9–11]. However, previous studies were conducted in surgical or mixed ICUs [4, 9–11]. In addition, they did not evaluate the relation between the development of ICU-acquired hyponatremia and relevant interventions. We hypothesized that medical ICU patients are more prone to be affected by dysnatremia and risk factors, considering more comorbidities and relative severity of illness requiring interventions in medical patients. Therefore, we investigated the incidence of ICU-acquired hyponatremia, the risk factors associated with its development, and its impact on patient outcomes in medical ICU.

## Methods

We conducted a retrospective cohort study based on the prospective registry of all critically ill patients admitted to the medical ICU of Samsung Medical Center, a 1989-bed university-affiliated, tertiary referral hospital in Seoul, South Korea, from January 1, 2015 to December 31, 2018. To be included, patients had to be 18 years or older and stay in the ICU for 48 h or more. For patients with multiple ICU admissions during the study period, only the first admission was selected for analysis. Patients who met the following criteria were excluded: (1) admission for postoperative care or neurological disorders with the exception of meningitis and metabolic coma, (2) hypo- or hypernatremia ([Na^+^] < 135 mmol/L or [Na^+^] > 145 mmol/L) at the time of ICU admission, (3) dependency on renal replacement therapy (RRT) at the time of ICU admission (i.e., patients who had been on dialysis or who were admitted to ICU for RRT), or (4) refusal to be registered in our database. For patients who experienced multiple sodium disturbance events, only the first dysnatremia event was analyzed.

The institutional review board of the Samsung Medical Center approved this study and waived the requirement for informed consent because of the observational nature of the research. Additionally, patient information was anonymized and de-identified prior to analysis.

### Data collection

Baseline characteristics at the time of admission, management variables during the first 3 days, and clinical outcomes were collected for each patient. Baseline characteristics include age, sex, body weight, Simplified Acute Physiology Score 3 (SAPS 3) and Sequential Organ Failure Assessment (SOFA) score, use of vasoactive agents and mechanical ventilation, reason for admission, underlying conditions, and initial laboratory values. Management profiles include daily input and output, medications known to affect sodium concentration [12], type of nutrition, and type and dose of blood products. Daily input was defined by the total volume administered by any route, such as intravenous injection or enteral provision. Daily output was defined by the total volume removed from the patient by any route, such as urine output or fluid drainage. Daily input and output over the first 3 days were added and then divided by the patient’s weight. Net volume balance was calculated by subtracting the output from the input. For patients who underwent RRT during their ICU stay, type and date of RRT initiation were also obtained. Data were extracted using our institution’s data repository (Clinical Data Warehouse Darwin-C, Samsung Medical Center, Seoul, Republic of Korea), which automatically retrieves data from electronic medical records.

Based on the previous reports on the ICU-acquired hyponatremia [4, 9–11], the primary outcome variable in this study was ICU-acquired hyponatremia. In addition, we collected final clinical outcomes: length of stay, ICU discharge status, and 28-day mortality after ICU admission.

### ICU-acquired hyponatremia

There are diverse criteria to define ICU-acquired hyponatremia in regard to reference sodium concentration, number of hyponatremia events, and timeframe for its onset [10, 13, 14]. In this study, we defined ICU-acquired hyponatremia as new-onset hyponatremia ([Na^+^] < 135 mmol/L) in patients whose sodium concentration was within the normal range (135 mmol/L ≤  [Na^+^] ≤ 145 mmol/L) at the time of ICU admission. We only evaluated events developed within 48 h after ICU admission in consideration of previous studies that have demonstrated a median time from ICU admission to onset of ICU-acquired hyponatremia of 2 days [10, 11].

### Statistical analysis

Continuous variables were reported as medians with interquartile ranges; categorical variables were reported as numbers and percentages. Eligible patients were assigned to either the ICU-acquired hyponatremia group or the normonatremia group, according to definition. Baseline characteristics and management variables were compared between the 2 groups using Fisher’s exact test and the Mann–Whitney U-test as appropriate. To find factors relevant to the development of ICU-acquired hyponatremia, a multivariate logistic regression was used incorporating variables with a *P* value less than 0.1 in univariate analysis and other variables deemed to be important by the authors. Based on the Akaike Information Criteria (AIC), a backward selection with a P < 0.05 for entry of variables and P > 0.10 for removal of variables was used to select variables for the multivariable model. The results were reported as odds ratios (OR) of each variable with 95% confidence intervals (CI). Finally, probability of RRT initiation for each group was estimated using the Kaplan–Meier method and compared using the log-rank test. Data were analyzed using SPSS (version 25.0; IBM, Armonk, NY). All tests were two-sided, and *P* values less than 0.05 were considered statistically significant.

## Results

**Fig. 1:**
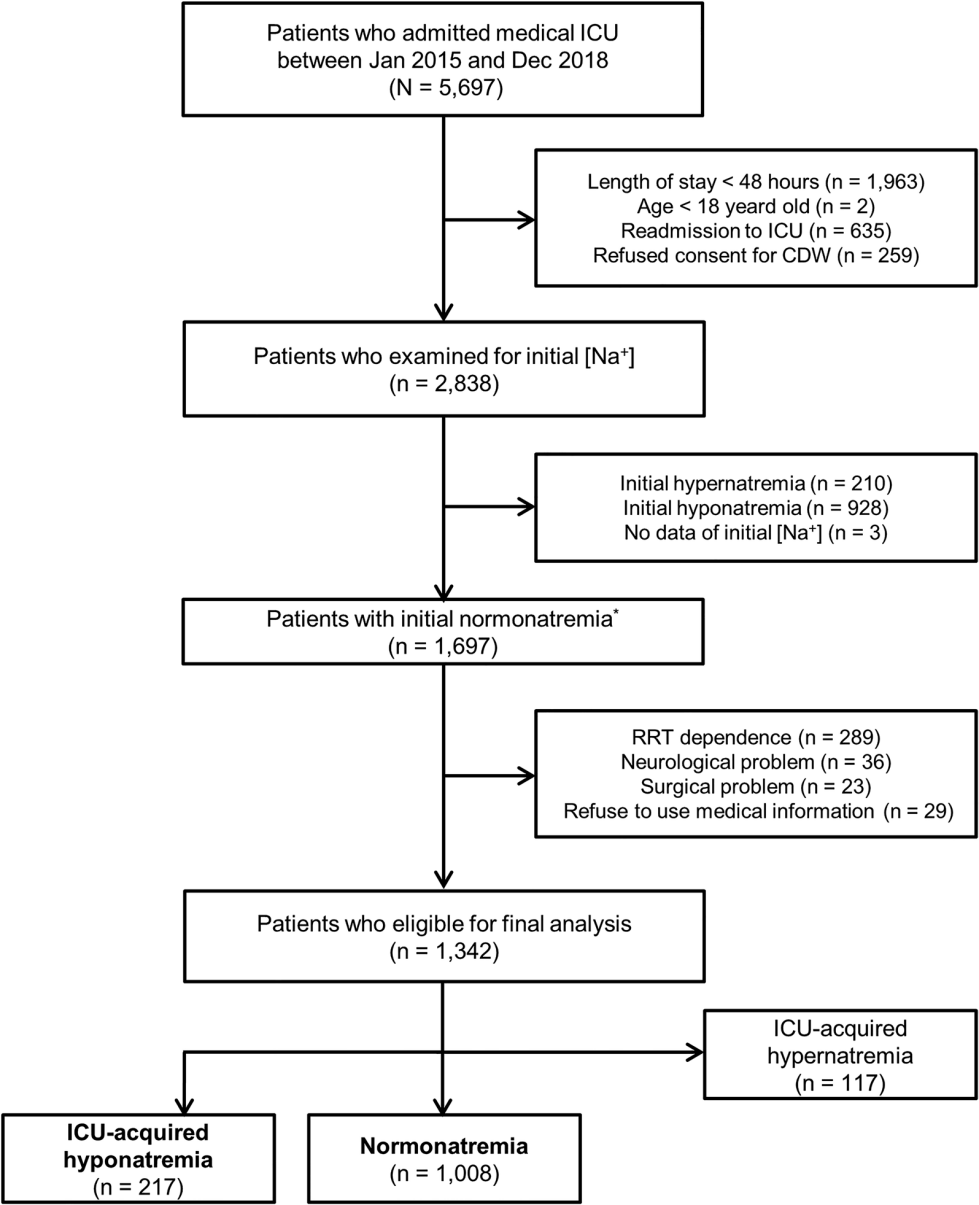
Study flow chart. *ICU* intensive care unit, *CDW* clinical data warehouse, *RRT* renal replacement therapy

During the study period, a total of 1697 patients with normonatremia at the time of ICU admission were eligible for this study. Of these, 289 (17.0%) patients who were dependent on RRT at the time of ICU admission, 59 (3.5%) patients with surgical or neurological problems, and 29 (1.7%) patients who refused use of their medical information were excluded. A total of 1342 eligible patients were identified based on our definition, including 1008 (75.1%) patients with normonatremia, 217 (16.2%) patients with ICU-acquired hyponatremia, and 117 (8.7%) patients with ICU-acquired hypernatremia (Fig. 1). To address the primary research objective of determining the factors associated with the development of ICU-acquired hyponatremia and clinical outcomes, patients with ICU-acquired hypernatremia were excluded from the final analysis.

### Baseline characteristics

The baseline characteristics of the 2 groups on ICU admission are shown in Table 1. Age and sex ratio were similar in both the hyponatnatremia and normonatremia groups. The median SOFA score (8.0 vs 7.0, *P* = 0.009) and SAPS3 (55.0 vs 51.0, *P* = 0.005) on ICU admission were significantly higher in the hyponatremia group. In addition, use of dobutamine (4.1% vs 1.8, *P* = 0.041) or vasopressin (17.1% vs 10.9%, *P* = 0.015) was more common in the hyponatremia group. However, reasons for ICU admission were similar except pulmonary disorder, which was more common in normonatremia group (40.6% vs 48.2%, *P* = 0.043). Comorbidities were also not different between the 2 groups, but chronic liver disease was more prevalent in the hyponatremia group (9.9% vs 5.3%, *P* = 0.030). Baseline levels of sodium (137.0 mmol/L vs 139.0 mmol/L, *P* < 0.001), potassium (4.2 mmol/L vs 4.0 mmol/L, *P* = 0.001), and creatinine (0.98 mg/dL vs 0.88 mg/dL, *P* = 0.034) were different between the two groups.

**Table 1:** Baseline characteristics

Characteristic	ICU-acquired hyponatremia (n = 217)	Normonatremia (n = 1008)	*P* value
Age, years	64.0 (53.0–73.0)	65.0 (55.0–74.0)	0.347
Male	141 (65.0)	617 (61.2)	0.317
SOFA	8.0 (5.0–10.0)	7.0 (4.0–10.0)	0.009
SAPS3	55.0 (44.5–67.0)	51.0 (41.0–63.0)	0.005
Organ support			
Vasoactive agent	76 (35.0)	336 (33.3)	0.635
Epinephrine	9 (4.1)	21 (2.1)	0.088
Norepinephrine	75 (34.6)	333 (33.0)	0.692
Dobutamine	9 (4.1)	18 (1.8)	0.041
Vasopressin	37 (17.1)	110 (10.9)	0.015
Mechanical ventilation	127 (58.5)	605 (60.0)	0.703
Reason for admission			
Cardiac	14 (6.5)	54 (5.4)	0.514
Pulmonary	88 (40.6)	486 (48.2)	0.043
Neurologic	4 (1.8)	14 (1.4)	0.542
Gastrointestinal	14 (6.5)	38 (3.8)	0.093
Sepsis	39 (18.0)	177 (17.6)	0.922
Other^a^	9 (4.1)	45 (4.5)	1.000
Comorbidity^b^			
IHD	13/160 (8.1)	54/778 (6.9)	0.613
Cardiomyopathy	6/160 (3.8)	19/777 (2.4)	0.415
Vascular disease	5/160 (3.1)	23/775 (3.0)	0.804
COPD	15/160 (9.4)	62/780 (7.9)	0.529
Asthma	3/160 (1.9)	20/777 (2.6)	0.783
ILD	5/160 (3.1)	41/779 (5.3)	0.317
Chronic liver disease	16/161 (9.9)	41/778 (5.3)	0.030
Hematologic malignancy	35/162 (21.6)	121/779 (15.5)	0.064
Oncologic malignancy	59/163 (36.2)	300/790 (38.0)	0.723
Na, mmol/L	137.0 (135.0–138.0)	139.0 (137.0–141.0)	< 0.001
K, mmol/L	4.2 (3.7–4.7)	4.0 (3.6–4.5)	0.001
BUN, mg/dL	22.3 (13.8–35.7)	21.0 (14.2–30.3)	0.140
Cr, mg/dL	0.98 (0.64–1.60)	0.88 (0.62–1.29)	0.034
GFR, mL/min/1.73 m^2^	72.8 (41.4–112.4)	81.9 (50.5–110.8)	0.067

Data are presented as number (percentage) or as median (interquartile range)

*ICU* intensive care unit, *SOFA* Sequential Organ Failure Assessment, *SAPS* Simplified Acute Physiology Score, *IHD* ischemic heart disease, *COPD* chronic obstructive pulmonary disease, *ILD* interstitial lung disease, *BUN* blood urea nitrogen, *GFR* glomerular filtration rate

^a^ Other includes drug intoxication, endocrine disorder, need for close monitoring or intensive nursing, environmental injuries, and experimental therapies with potential complications

^b^ Because of missing data, the available number of patients is presented as a denominator

### Management during the first 3 days

Management profiles during the first 3 days after ICU admission are shown in Table 2. Median net volume balance over the first 3 days was significantly higher in the hyponatremia group (19.4 mL/kg vs 11.5 mL/kg, *P* = 0.004). The median input and output were also significantly higher in the hyponatremia group. For medications known to cause sodium disturbance, sodium bicarbonate was more commonly used in the hyponatremia group (27.2% vs 18.3%, *P* = 0.004).

**Table 2:** Management profiles during the first 3 days

Management profiles	ICU-acquired hyponatremia (n = 217)	Normonatremia (n = 1008)	*P* value
Volume balance^a^, mL/kg			
Net	19.4 (−13.6–67.7)	11.5 (−20.8–44.9)	0.004
Input	161.4 (117.3–245.0)	141.0 (93.6–196.7)	< 0.001
Output	144.9 (90.4–211.7)	125.0 (86.2–178.1)	0.016
Medication			
Diuretics	150 (69.1)	675 (67.0)	0.577
Furosemide	149 (68.7)	669 (66.4)	0.526
Thiazide	2 (0.9)	12 (1.2)	1.000
Spironolactone	10 (4.6)	45 (4.5)	0.858
Sodium bicarbonate	59 (27.2)	184 (18.3)	0.004
Mannitol	1 (0.5)	1 (0.1)	0.323
Nutrition			
Enteral nutrition	59 (27.2)	310 (30.8)	0.328
Parenteral nutrition	30 (13.8)	168 (16.7)	0.360
Transfusion			
RBC	18 (8.3)	73 (7.2)	0.570
Platelet	217 (23.5)	202 (20.0)	0.267
FFP	32 (14.7)	96 (9.5)	0.027

Data are presented as number (percentage) or as median (interquartile range)

*ICU* intensive care unit, *RBC* red blood cell, *FFP* fresh frozen plasma

^a^Total input and output from day 0 (ICU admission) to day 2 were combined and divided by body weight

Use and types of diuretics were not different between the two groups. Fresh frozen plasma (FFP) was more frequently administered in the hyponatremia patients (14.7% vs 9.5%, *P* = 0.027).

### Factors associated with ICU-acquired hyponatremia

Table 3 lists the results of both univariate and multivariate analysis of factors associated with the development of ICU-acquired hyponatremia. Among baseline characteristics, hematologic malignancy (adjusted OR, 1.692; 95% CI 1.076–2.663; *P* = 0.023) and initial potassium concentration (adjusted OR, 1.637; 95% CI 1.296–2.069; *P* < 0.001) were independently associated with the development of ICU-acquired hyponatremia. In addition, net volume balance was the only management profile significantly associated with ICU-acquired hyponatremia (adjusted OR, 1.004; 95% CI 1.002–1.007; *P* = 0.001).

**Table 3:** Factors associated with ICU-acquired hyponatremia

Factors	Univariable analysis		Multivariable analysis^*^	
	Crude OR (95% CI)	*P* value	Adjusted OR (95% CI)	*P* value
Age	0.995 (0.986–1.004)	0.302		
Male	1.176 (0.865–1.597)	0.300		
SOFA	1.052 (1.012–1.094)	0.010		
SAPS3	1.012 (1.003–1.021)	0.012		
Mechanical ventilation	0.940 (0.698–1.266)	0.684		
Vasoactive agent	1.078 (0.792–1.467)	0.633		
Reason for admission				
Cardiac	1.218 (0.664–2.236)	0.524		
Pulmonary	0.733 (0.544–0.987)	0.041		
Neurologic	1.333 (0.435–4.091)	0.615		
Gastrointestinal	1.760 (0.937–3.309)	0.079		
Sepsis	1.029 (0.702–1.508)	0.885		
Others	0.926 (0.460–1.924)	0.837		
Underlying condition				
IHD	1.186 (0.631–2.228)	0.597		
Cardiomyopathy	1.554 (0.611–3.955)	0.355		
Vascular disease	1.055 (0.395–2.817)	0.915		
COPD	1.198 (0.663–2.164)	0.549		
Asthma	0.723 (0.212–2.464)	0.604		
ILD	0.581 (0.226–1.493)	0.259		
Chronic liver disease	1.984 (1.084–3.631)	0.026		
Hematologic malignancy	1.499 (0.983–2.284)	0.060	1.692 (1.076–2.663)	0.023
Oncologic malignancy	0.927 (0.653–1.315)	0.670		
Na	0.734 (0.685–0.787)	< 0.001		
K	1.466 (1.216–1.768)	< 0.001	1.637 (1.296–2.069)	< 0.001
BUN	1.012 (1.003–1.020)	0.009		
Cr	1.304 (1.116–1.524)	0.001		
GFR	0.997 (0.995–1.000)	0.079		
Net fluid balance	1.004 (1.002–1.006)	< 0.001	1.004 (1.002–1.007)	0.001
Any diuretics	1.104 (0.805–1.516)	0.538		
Furosemide	1.110 (0.810–1.522)	0.515		
Thiazide	0.772 (0.172–3.475)	0.736		
Spironolactone	1.034 (0.513–2.085)	0.926		
Sodium bicarbonate	1.672 (1.191–2.347)	0.003		
Mannitol	4.662 (0.290–74.825)	0.277		
Enteral nutrition	0.841 (0.606–1.167)	0.300		
Parenteral nutrition	0.802 (0.527–1.220)	0.303		
RBC	1.159 (0.676–1.984)	0.592		
Platelet	1.226 (0.864–1.739)	0.254		
FFP	1.643 (1.069–2.526)	0.024		

*OR* odds ratio, *CI* confidence interval, *SOFA* Sequential Organ Failure Assessment, *SAPS* Simplified Acute Physiology Score, *IHD* ischemic heart disease, *COPD* chronic obstructive pulmonary disease, *ILD* interstitial lung disease, *BUN* blood urea nitrogen, *GFR* glomerular filtration rate, *RBC* red blood cell, *FFP* fresh frozen plasma

^*^ Variables with a *P* value less than 0.1 in univariable analysis and other variables deemed to be important by authors (mechanical ventilation, vasopressor, age, and sex) were incorporated to multivariable analysis. Although BUN, Cr and GFR were all significant in univariate analysis, only GFR was used in multivariate analysis

### Clinical outcomes

**Fig. 2:**
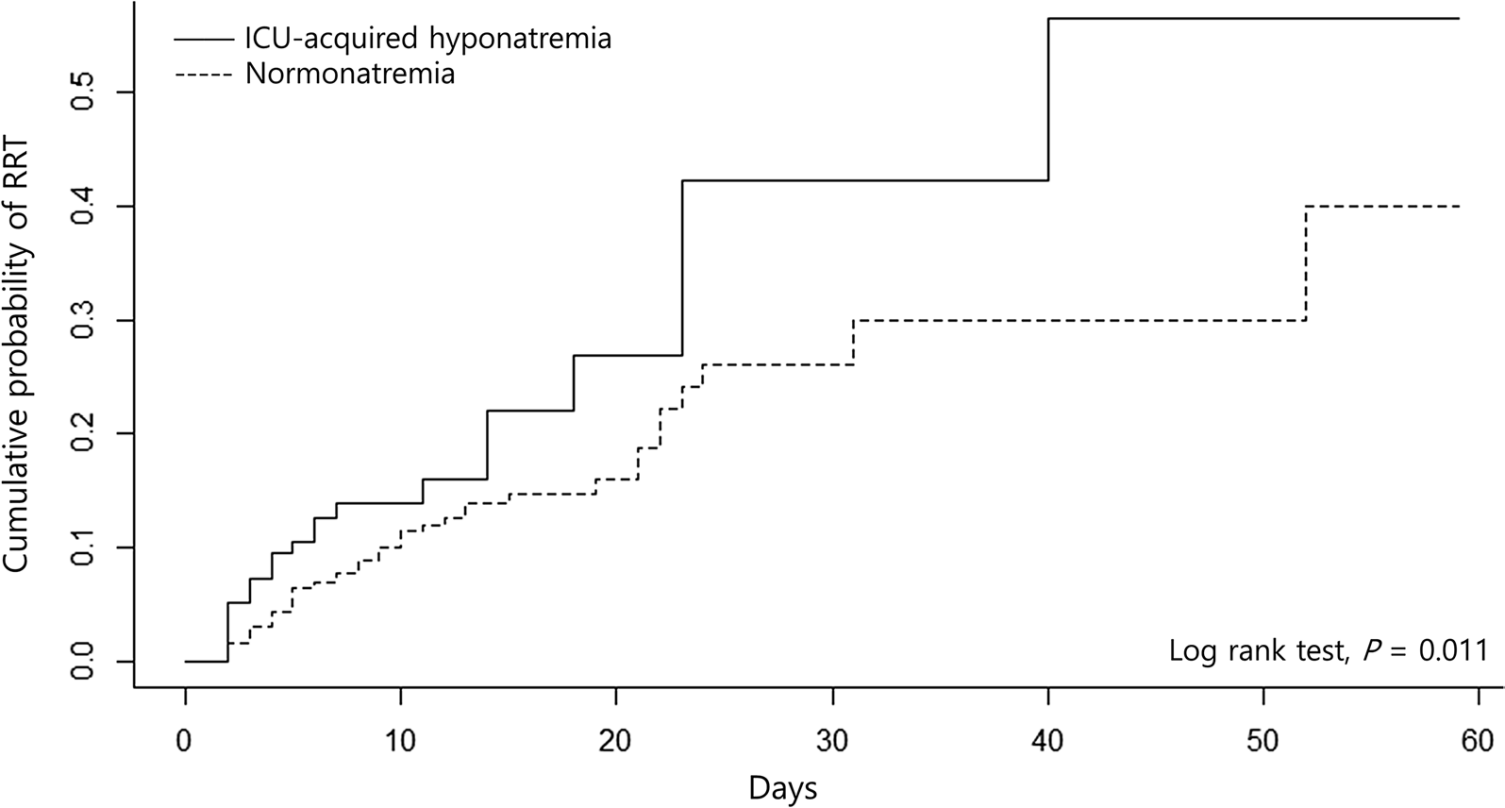
Kaplan–Meier curves showing the probability of initiation of renal replacement therapy. *ICU* intensive care unit, *RRT* renal replacement therapy

Comparisons of clinical outcomes for the two groups are presented in Table 4. ICU mortality (15.2% vs 14.4%, *P* = 0.751) and 28-day mortality (20.7% vs 21.3%, *P* = 0.927) were similar between the two groups. The median ICU LOS was not different between the 2 groups (*P* = 0.216). A total of 104 (12.7%) patients were supported with RRT after ICU admission, and patients with ICU-acquired hyponatremia were more likely to require RRT (13.4% vs 7.4%, *P* = 0.007). The time to initiate RRT was not different in the 2 groups (3.0 days vs 5.0 days, *P* = 0.262), but the risk of RRT was consistently higher in ICU-acquired hyponatremia group during their ICU stay (Fig. 2).

**Table 4:** Clinical Outcomes

Outcomes	ICU-acquired hyponatremia (n = 217)	Normonatremia (n = 1008)	*P* value
ICU mortality	33 (15.2)	145 (14.4)	0.751
28-day mortality	45 (20.7)	215 (21.3)	0.927
ICU length of stay	5.3 (3.0–10.9)	4.9 (3.0–9.3)	0.216
Renal replacement therapy^a^	29 (13.4)	75 (7.4)	0.007
CRRT	25 (11.5)	70 (6.9)	0.035
Intermittent hemodialysis	9 (4.1)	16 (1.6)	0.029
Time to renal replacement therapy (days)	3.0 (2.0–9.0)	5.0 (3.0–9.0)	0.262

Data are presented as number (percentage) or as median (interquartile range)

*ICU* intensive care unit, *CRRT* continuous renal replacement therapy

^a^Some patients were supported by both continuous renal replacement therapy and intermittent hemodialysis

## Discussion

In the present study, we found that 16.2% of critically ill medical patients who had normal sodium concentration at the time of ICU admission developed new-onset hyponatremia within the first 48 h after admission and that net volume balance was the only modifiable factor associated with ICU-acquired hyponatremia. In addition, ICU-acquired hyponatremia patients were more likely to require RRT, although other clinical outcomes were not different between the two groups.

Contrary to our expectation that hyponatremia would be more prevalent in medical ICU patients, the incidence of ICU-acquired hyponatremia in this study was not higher compared with previous studies [4, 9–11]. This finding should be interpreted cautiously because we only evaluated events that developed within first 48 h, whereas previous studies investigated the entire duration of the ICU stay [4, 9–11]. Therefore, it would be expected that incidence would be increased if the time frame was extended.

Stelfox et al. demonstrated the inverse relationship between ICU-acquired hyponatremia and hyperkalemia as a time-dependent factor [10, 11]. Our study also indicates that ICU-acquired hyponatremia was more prevalent in patients with higher potassium concentration on ICU admission. Furthermore, the exact opposite relationship was shown between initial hypokalemia and ICU-acquired hypernatremia [15]. These findings suggest that a single measurement of potassium concentration on ICU admission could be helpful in predicting subsequent ICU-acquired hyponatremia.

Hematologic malignancy was the only underlying condition related to the development of ICU-acquired hyponatremia. Contrary to our results, the prevalence of hematologic malignancy was lower in patients with ICU-acquired hyponatremia in the previous two studies, but there was no statistical significance [4, 9]. It is well known that hyponatremia and malignancy are closely related [16], and several factors were suggested as contributors to hyponatremia, including antineoplastic therapy, adrenal metastasis, and arginine vasopressin [17]. However, these are insufficient to explain our findings because patients with pre-existing hyponatremia were excluded and patients with oncologic malignancy were evenly distributed in this study. In addition, 16.6% of our study population had hematologic malignancy, which is much higher than the proportion of hematologic malignancy in previous studies [4, 9]. This large number of patients suggests that it is not likely to be obtained by chance. Additional research is needed to ascertain the association between hematologic malignancy and ICU-acquired hyponatremia.

We investigated several management profiles to determine whether there are modifiable factors that give rise to ICU-acquired hyponatremia. We found that use of sodium bicarbonate and FFP, as well as net volume balance, were different between the 2 groups. Of these, net volume balance was the only variable of significance in multivariate analysis. This result is in agreement with the study by Steiglmair et al. showing that positive fluid balance was a single reason of ICU-acquired hyponatremia in 25% of cardiothoracic surgery patients [13]. Interestingly, although not proven in multivariate analysis, use of sodium bicarbonate, which is an established risk factor of hypernatremia, was associated with ICU-acquired hyponatremia [12]. It is tempting to speculate that frequent administration of sodium bicarbonate reflects metabolic acidosis in patients with acute kidney injury. This is supported by the result that patients with ICU-acquired hyponatremia were more frequently treated with RRT than normonatremia patients. The same result has been reported in patients after cardiac surgery [11], and new-onset hyponatremia was also related to acute kidney injury and in postoperative patients [18]. It is uncertain whether ICU-acquired hyponatremia and renal failure coexist independently in severe patients or if ICU-acquired hyponatremia precedes manifestation of worsening renal function. Unfortunately, our data could not establish a causal relationship because of inherent limitation of observational design of the study. However, our data suggest that renal function should be closely monitored in patients with ICU-acquired hyponatremia based on the significant association between hyponatremia and renal dysfunction.

Considering the comorbidities and relative severity of illness in medical patients admitted to ICUs, limited data is available on whether ICU-acquired hyponatremia in medical ICU patients is associated with poor clinical outcomes. In the present study, clinical outcomes such as ICU mortality or LOS in patients with ICU-acquired hyponatremia was not worse compared with patients with normonatremia, inconsistent with previous reports [4, 9–11]. In a large retrospective study with a database of 8142 adults admitted to 3 medical-surgical ICUs consecutively over a period of 6 years [10], Stelfox et al. demonstrated that ICU-acquired hyponatremia and its grade were associated with increased ICU mortality. However, the proportion of medical patients included in the study was only 44%, which limits representation of clinical outcomes in medical patients with ICU-acquired hyponatremia. The other studies included surgical patients only [4, 9, 11], in which ICU mortality is relatively lower than medical patients. Therefore, further studies with a larger number of medical patients are needed to substantiate our findings.

Although this study provided new information on the factors associated with ICU-acquired hyponatremia in medical ICUs, there are several limitations that should be acknowledged. First, given the observational nature of this study, selection bias may have influenced the significance of its findings. This was addressed by adjusted multivariate analysis. However, the potential for bias due to an unmeasured confounder remains. Furthermore, the study was conducted at a single institution with a specialized ICU for critically ill cancer patients, resulting in a high proportion of cancer patients in the study, which may limit the generalizability of our findings. Second, we could not collect detailed information on type and amount of intravenous fluid, dose of each medication, and constituents and amount of nutrition therapy. These factors might have an influence on electrolyte and water imbalance, however, which could not be extracted from our data repository. Third, we only evaluated events that occurred during first 48 h, which could alter some findings. However, 50% of ICU-acquired hyponatremia developed within 2 days after admission [10], and confounding factors would increase as the length of the ICU stay increased. Therefore, we evaluated only events in first 48 h to evaluate modifiable risk factors during the early phase of ICU admission.

## Conclusion

In conclusion, ICU-acquired hyponatremia is not uncommon in critically ill medical patients. Our findings suggest a possible link between ICU-acquired hyponatremia and patient management, especially net volume balance and use of renal replacement therapy but not to mortality.

## Data Availability

The data that support the findings of this study are available on request from the corresponding author. The data are not publicly available due to privacy or ethical restrictions.

## Abbreviations

CI: Confidence interval
FFP: Fresh frozen plasma
ICU: Intensive care unit
LOS: Length of stay
OR: Odds ratio
RRT: Renal replacement therapy
SAPS: Simplified Acute Physiology Score
SOFA: Sequential Organ Failure Assessment
